# Plasminogen activator inhibitor 1 promotes aortic aging–like pathophysiology in humans and mice

**DOI:** 10.1172/JCI196714

**Published:** 2025-09-30

**Authors:** Alireza Khoddam, Anthony Kalousdian, Mesut Eren, Saul Soberanes, Andrew Decker, Elizabeth J. Lux, Benjamin W. Zywicki, Brian Dinh, Bedirhan Boztepe, Baljash S. Cheema, Carla M. Cuda, Hiam Abdala-Valencia, Arun Sivakumar, Toshio Miyata, Lisa D. Wilsbacher, Douglas E. Vaughan

**Affiliations:** 1 Feinberg Cardiovascular and Renal Research Institute,; 2 Department of Medicine, Northwestern University Feinberg School of Medicine, Chicago, Illinois, USA.; 3 Tohoku University Graduate School of Medicine, Sendai, Japan.

**Keywords:** Cardiology, Genetics, Vascular biology, Cardiovascular disease, Serpins, Transcriptomics

## Abstract

Plasminogen activator inhibitor 1 (PAI-1), encoded by *SERPINE1*, contributes to age-related cardiovascular disease (CVD) and other aging-related pathologies. Humans with a heterozygous loss-of-function *SERPINE1* variant exhibit protection against aging and cardiometabolic dysfunction. We engineered a mouse model mimicking the human mutation (*Serpine1^TA700/+^*) and compared cardiovascular responses with WT littermates. *Serpine1*^TA700/+^ mice lived 17% longer than did littermate control mice. Under l-N^G^-nitro-arginine methyl ester–induced (l-NAME–induced) vascular stress, *Serpine1^TA700/+^* mice exhibited diminished pulse wave velocity (PWV), lower systolic blood pressure (SBP), and preserved left ventricular diastolic function compared with controls. Conversely, PAI-1–overexpressing mice had measurements indicating accelerated cardiovascular aging. Single-cell transcriptomics of *Serpine1^TA700/+^* aortas revealed a vascular-protective mechanism with downregulation of the extracellular matrix regulators *Ccn1* and *Itgb1*. *Serpine1^TA700/+^* aortas were also enriched in a cluster of smooth muscle cells that exhibited plasticity. Finally, PAI-1 pharmacological inhibition normalized SBP and reversed l-NAME–induced PWV elevation. These findings demonstrate that PAI-1 reduction protects against cardiovascular aging-related phenotypes, while PAI-1 excess promotes vascular pathological changes. Taken together, PAI-1 inhibition represents a promising strategy to mitigate age-related CVD.

## Abstract 2

This study demonstrates that Plasminogen activator inhibitor-1 (PAI-1) is both necessary and sufficient for vascular aging, and that inhibition of PAI-1 reverses aging-related vascular pathology.

## Graphical abstract

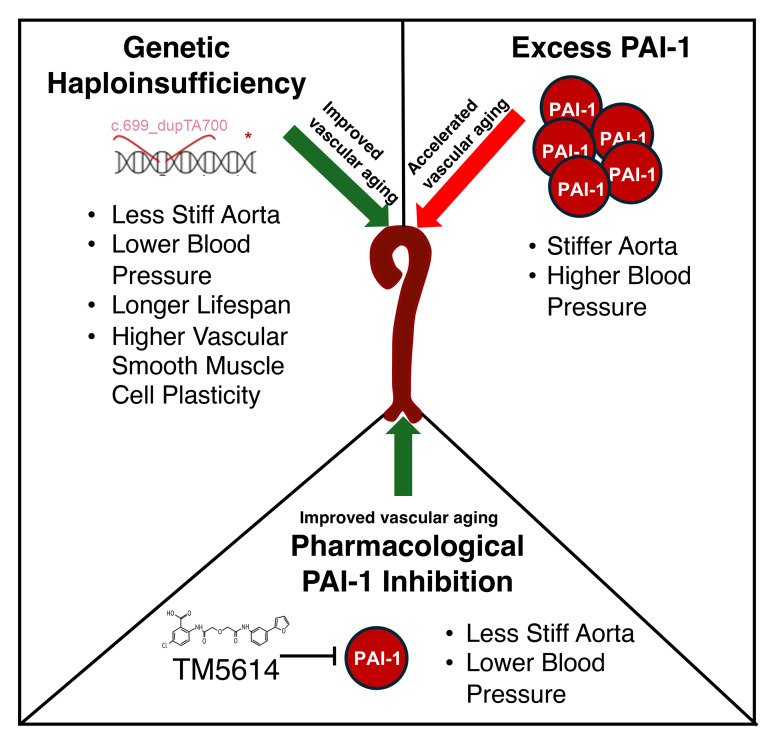

## Introduction

Plasminogen activator inhibitor 1 (PAI-1) is a critical contributor in age-related cardiovascular disease (CVD) (1, 2). PAI-1 was originally identified as an inhibitor of tissue-type plasminogen activator (t-PA) and urokinase plasminogen activator (u-PA), and therefore it promotes vascular thrombosis (3). However, subsequent studies revealed that PAI-1 also contributes to senescence: senescent cells secrete higher PAI-1 levels as part of the senescence-associated secretory phenotype (SASP), accelerating the accumulation of additional senescent cells (4); furthermore, PAI-1 is also a necessary and sufficient mediator of senescence (4–6). Additional work in mice demonstrated that PAI-1 also affects aging (7). Clinically, elevated PAI-1 levels in humans correlate with coronary artery disease (8, 9), increased vascular stiffness (10), obesity (11, 12), and diabetes (13). Methylation-based assessment of PAI-1 expression levels consistently predicts earlier time to mortality (14). Experimental studies link PAI-1 to arteriosclerosis (15), age-dependent coronary thrombosis (16, 17), endothelial dysfunction (18), and disruptions of lipid metabolism (19). These findings suggest that PAI-1 is a potential target for combating age-related cardiovascular decline.

Pharmacological inhibition of PAI-1 has gained momentum because of its pleiotropic role in age-related diseases (20). Small-molecule inhibitors have been developed to reduce PAI-1 activity, with evidence in animal models showing improvements in vascular function (21), reduced thrombosis (22), and amelioration of metabolic dysfunction (23). In humans, a rare dinucleotide duplication mutation in the *SERPINE1* gene (designated *SERPINE1*c.699_700dupTA) was identified as a founder mutation in a Swiss Amish population in Berne, Indiana (USA). This loss-of-function (LOF) mutation leads to a dose-dependent reduction in plasma PAI-1 levels (both activity and antigen) (24). Individuals with LOF heterozygosity exhibit longer leukocyte telomere lengths, lower fasting insulin levels, and reduced diabetes prevalence (25). This “heterozygous advantage” is also characterized by an extension of the human lifespan by a median of 10 years (25).

The risk of CVD increases with age (26, 27), and this risk culminates with CVD as the leading cause of death worldwide (28). Vascular stiffness accelerates CVD development, especially in aging populations, by damaging the microvasculature of organs like the brain and kidneys; elevated vascular stiffness increases the risk of hypertension, atherosclerosis, heart attacks, cognitive decline, and strokes (29). Arterial stiffness, measured physiologically by pulse wave velocity (PWV), is well known to rise with age (30–34); notably, increases in PWV precede overt hypertension and CVD (31–33), and PWV has been described as a tool to measure vascular aging (31). Recent work suggested that vascular aging is one of the primary drivers of systemic aging (35) and a major contributor to the development of various other age-related diseases. Cellularly, endothelial cells regulate vascular tone and vasodilation via nitric oxide (NO) production by endothelial NO synthase (eNOS) (36). Aging in murine models has been linked to increased uncoupling of the eNOS complex (37), and PAI-1 was recently identified as a negative regulator of eNOS, impairing NO production in vitro (18). This in vitro finding partly explains the cardiovascular and longevity benefits of PAI-1 reduction. Thus, we hypothesize that reducing circulating PAI-1 levels protects against vascular stiffness and vascular aging.

In this report, we investigated the role of PAI-1 on cardiovascular physiology using genetically engineered mouse models. We reverse-engineered a mouse line carrying a dinucleotide duplication mutation in *Serpine1* exon 4 (*Serpine1^c.699_700dupTA^*), mirroring the human LOF mutation associated with protection against biological aging. We subsequently treated heterozygous mutated mice (hereafter referred to as *Serpine1^TA700/+^* mice) with an eNOS inhibitor to model aging-related vascular stiffening and increased PWV. Additionally, we used another mouse model overexpressing stabilized human PAI-1 (16) to compare PWV changes with aging. Bulk and single-cell RNA-Seq (scRNA-Seq) identified candidate genes associated with the protective effects of PAI-1 reduction. Last, we tested the ability of a small-molecule PAI-1 inhibitor to reverse damages to the vasculature via eNOS inhibition.

## Results

### Heterozygous SERPINE1 deficiency confers vascular fitness in humans.

**Figure 1:**
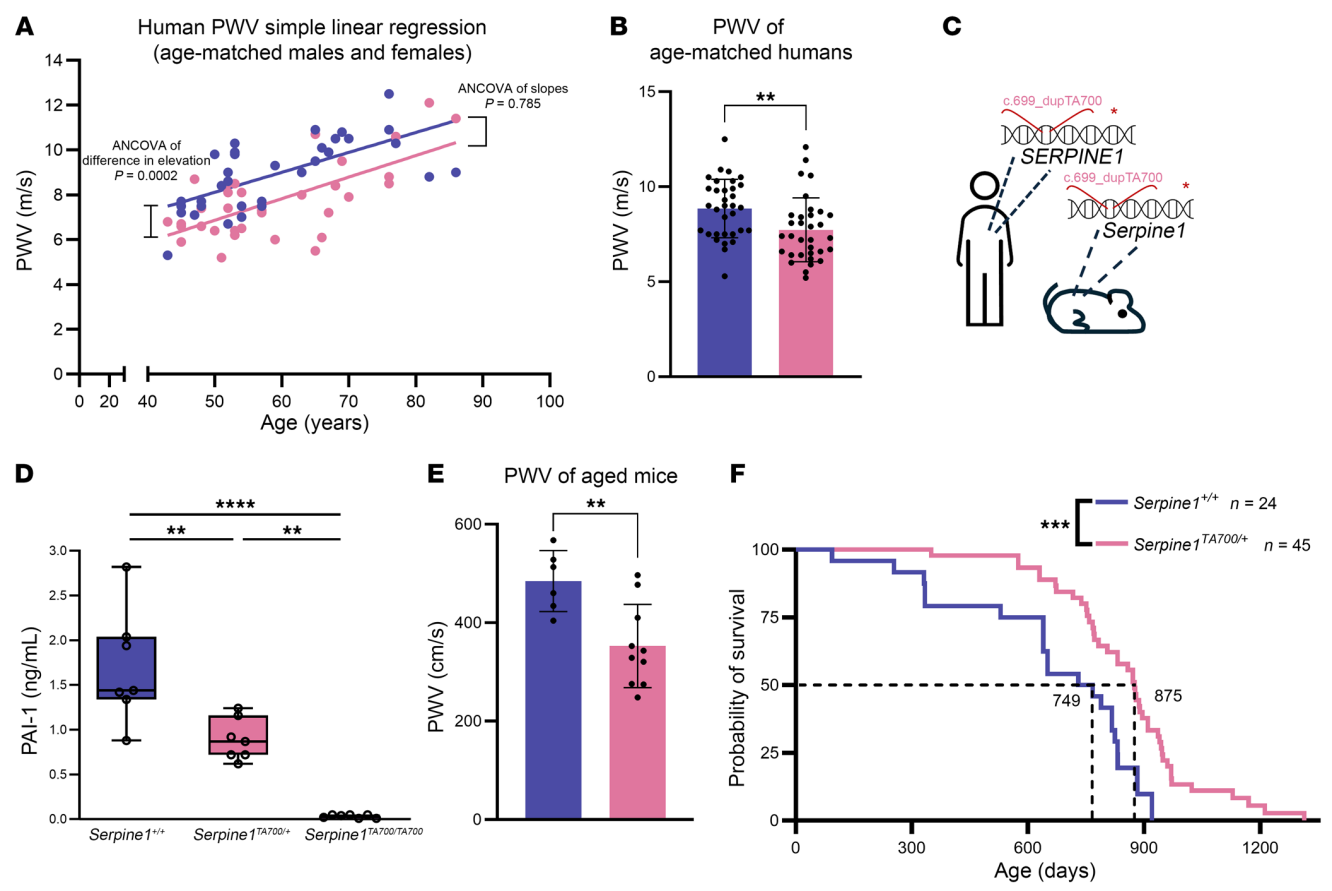
*SERPINE1* haploinsufficiency is associated with improved vascular function in humans and longer lifespan in mice. (**A**) Scatter plot of PWV as a function of age in age- and sex-matched individuals with *SERPINE1^TA700/+^* (*n* = 33, 11 male and 22 female participants) or *SERPINE1^+/+^* (*n* = 33, 11 male and 22 female participants) genotypes. Simple linear regression was used to assess the PWV as a function of age. (**B**) Aggregated human PWV values. (**C**) Schematic of the mouse line harboring a dinucleotide duplication in the mouse gene *Serpine1*. (**D**) Serum PAI-1 levels measured by detecting PAI-1 antigen using ELISA (*n* = 7 per genotype). (**E**) Comparison of aged (600 days and older) *Serpine1^+/+^* (*n* = 6: 4 males and 2 females) and aged (600 days and older) *Serpine1^TA700/+^* (*n* = 10: 6 males and 4 females) mice. (**F**) Overall survival of *Serpine1^TA700/+^* and control *Serpine1^+/+^* mice (*n* = 9 male and *n* = 15 female *Serpine1^+/+^* mice; *n* = 27 male and *n* = 18 female *Serpine1^TA700/+^* mice). **P < 0.01, ***P < 0.001, and ****P < 0.0001, by ANCOVA (**A**), 2-tailed, unpaired *t* test (**B** and **E**), ordinary 1-way ANOVA (**D**), and a log-rank test (**F**). Blue indicates *SERPINE1^+/+^* for humans and *Serpine1^+/+^* for mice, and pink indicates *SERPINE1^TA700/+^* for humans and *Serpine1^TA700/+^* for mice.

To build on the initial findings in the Swiss Amish population in Berne, Indiana (25), we recruited additional individuals from this population and expanded the phenotyping protocol to include PWV. We compared individuals carrying the *SERPINE1* c.699_700dupTA mutation (*SERPINE1^TA700/+^*, *n* = 33, with 11 male and 22 female participants) with sex- and age-matched unaffected controls (*SERPINE1^+/+^*, *n* = 33, with 11 male and 22 female participants). When adjusting for age and sex, we found that PWV values were significantly lower in the *SERPINE1^TA700/+^* group, suggesting a potential protective effect against vascular stiffening (Table 1). The parameter estimate for genotype was –1.182 m/s (95% CI: –1.784 to –0.580, *P* = 0.0002), indicating that, after adjusting for age and sex, *SERPINE1^TA700/+^* carriers had PWV values that were, on average, 1.182 m/s lower than those of noncarriers. Given the clinical observations, this magnitude of reduction is linked to a meaningful decrease in all-cause mortality risk (38). Using the same data from this cohort, we performed simple linear regression and found a positive correlation between age and PWV. The slope of each line did not differ between genotypes; however, elevation of the line was lower in *SERPINE1^TA700/+^* carriers as compared with *SERPINE1^+/+^* individuals, as expected from our adjusted analyses (Figure 1A). We also analyzed the aggregated PWV averages of individuals and found that PWV was roughly 12% lower in *SERPINE1^TA700/+^* carriers than in noncarriers (Figure 1B). Together, these statistical analyses confirm that PWV increased with age in both genotypes at a comparable rate; however, the *SERPINE1^TA700/+^* variant was associated with an overall lower PWV, even after adjusting for age. Stratifying the PWV values by biological sex yielded consistent results, with statistically significant differences observed in both male and female individuals (Supplemental Figure 1; supplemental material available online with this article; https://doi.org/10.1172/JCI196714DS1). Our findings prompted us to dissect the mechanisms underlying this vascular improvement using experimental animal models.

### Serpine1^TA700/+^ mice have a longer lifespan and reduced aging-related aortic stiffness.

To examine potential mechanisms by which the LOF *SERPINE1^c.699_700dupTA^* mutation confers protection against biological aging (25), we utilized CRISPR-based gene editing on C57BL6/J mice to insert a TA dinucleotide at the end of exon 4 of the *Serpine1* gene (Figure 1C and Supplemental Figure 2). We measured PAI-1 levels in circulating plasma from mutated mice using ELISA. *Serpine1^TA700/+^* and *Serpine1^TA700/TA700^* exhibited a 50% and 100% reduction, respectively, in circulating PAI-1 antigen levels compared with *Serpine1^+/+^* controls (Figure 1D). We measured the PWV of *Serpine1^TA700/+^* and control mice that were older than 85 weeks of age. We found that *Serpine1^TA700/+^* mice had roughly 20% lower PWV compared with *Serpine1^+/+^* mice (Figure 1E). Akin to the longer lifespan phenotype observed in humans (25), *Serpine1^TA700/+^* mice had 17% longer overall survival compared with WT littermates (Figure 1F). The length of lifespan was not sex dependent between male and female *Serpine1^TA700/+^* mice (Supplemental Figure 3).

### Serpine1 haploinsufficiency protects against l-NAME–induced aging-like pathophysiology.

**Figure 2:**
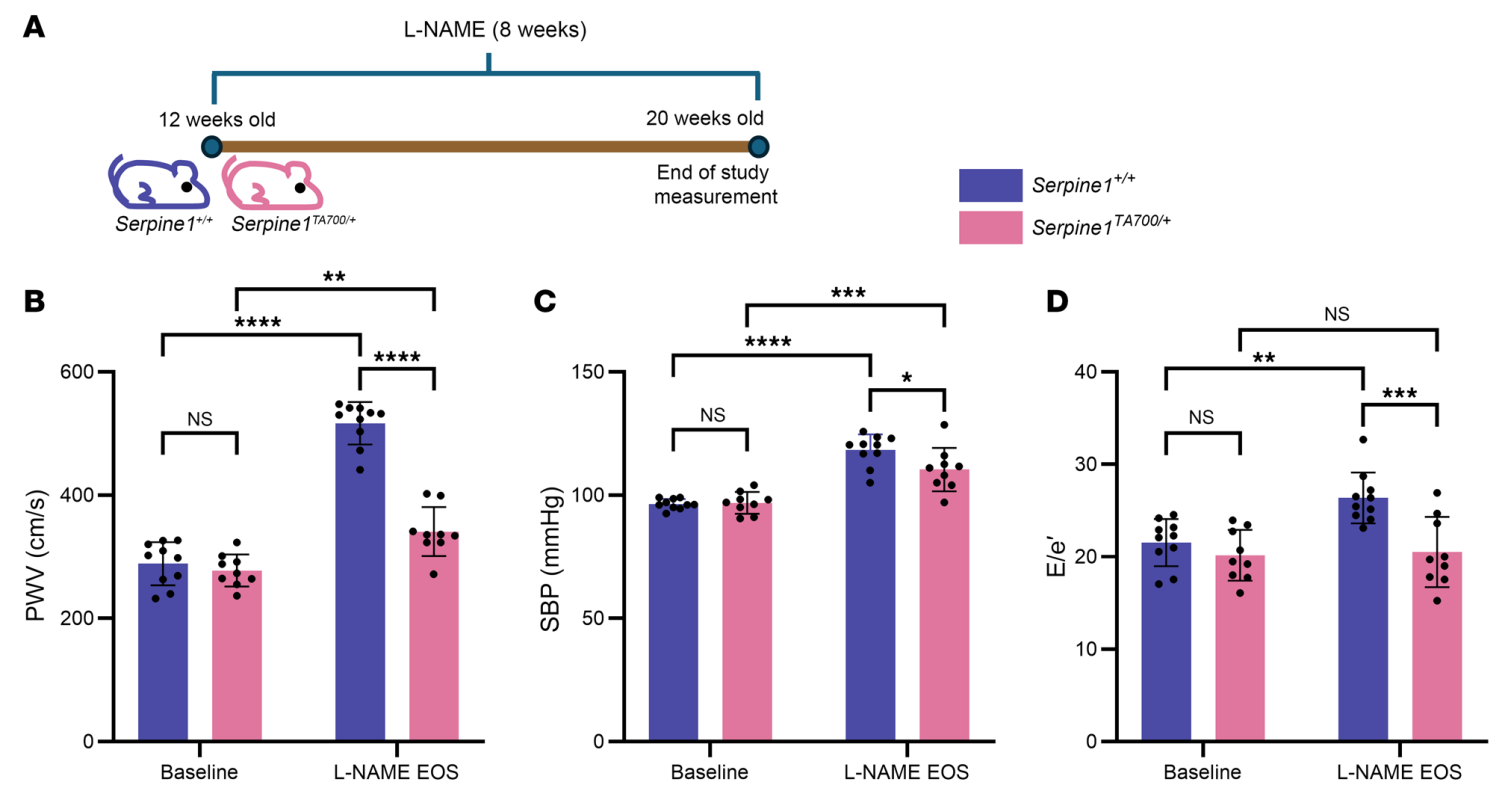
*Serpine1^TA700/+^* mice exhibit protection against cardiovascular pathophysiology associated with l-NAME. (**A**) Schematic of the study: *n* = 10 *Serpine1^+/+^* mice (7 males and 3 females); *n* = 9 *Serpine1^TA700/+^* mice (6 males and 3 females). (**B**) PWV (**C**) SBP, and (**D**) E/e′ for *Serpine1^TA700/+^* mice compared with littermate controls at baseline and at the end of the study (EOS). *P < 0.05, **P < 0.01, ***P < 0.001, and ****P < 0.0001, by 2-way ANOVA was used in (**B**–**D**) for significance between variables.

We hypothesized that the reduction of PAI-1 in *Serpine1^TA700/+^* would protect the mice against cardiovascular stress. To model hypertension associated with aging, we treated *Serpine1^TA700/+^* mice and their control littermates with l-N^G^-nitro-arginine methyl ester (l-NAME) (Figure 2A). This established model inhibits eNOS, causing endothelial dysfunction (39), increased senescence (21), arterial stiffness (40), and hypertension (41). We quantified changes associated with l-NAME–induced stress using physiological measures, including descending aorta PWV, systolic blood pressure (SBP), and diastolic function (E/e′). At baseline, PWV, SBP, and E/e′ were comparable between *Serpine1*^TA700/+^ mice and control littermates (Figure 2, B–D). Following 8 weeks of l-NAME treatment, we found that the increase in PWV, SBP, and E/e′ was significantly attenuated in *Serpine1^TA700/+^* mice compared with control littermates (Figure 2, B–D). By stratifying measurements according to biological sex, we confirmed consistent and statistically significant differences across groups, with only the end-of-study SBP in male *Serpine1^TA700/+^* mice not reaching statistical significance (Supplemental Figure 4). Furthermore, the increase in PWV with an 8-week period of l-NAME treatment in 3-month-old mice for both *Serpine1^+/+^* and *Serpine1^TA700/+^* animals was comparable to the PWV values for aged mice (Supplemental Figure 5); this observation provides evidence that l-NAME treatment recapitulated the aging-like vascular pathologies associated with chronological aging as measured using PWV. These data suggest that PAI-1 is necessary to promote the aging-like pathophysiological changes induced by l-NAME.

### PAI-1 overexpression exacerbates aging-like pathophysiology.

**Figure 3:**
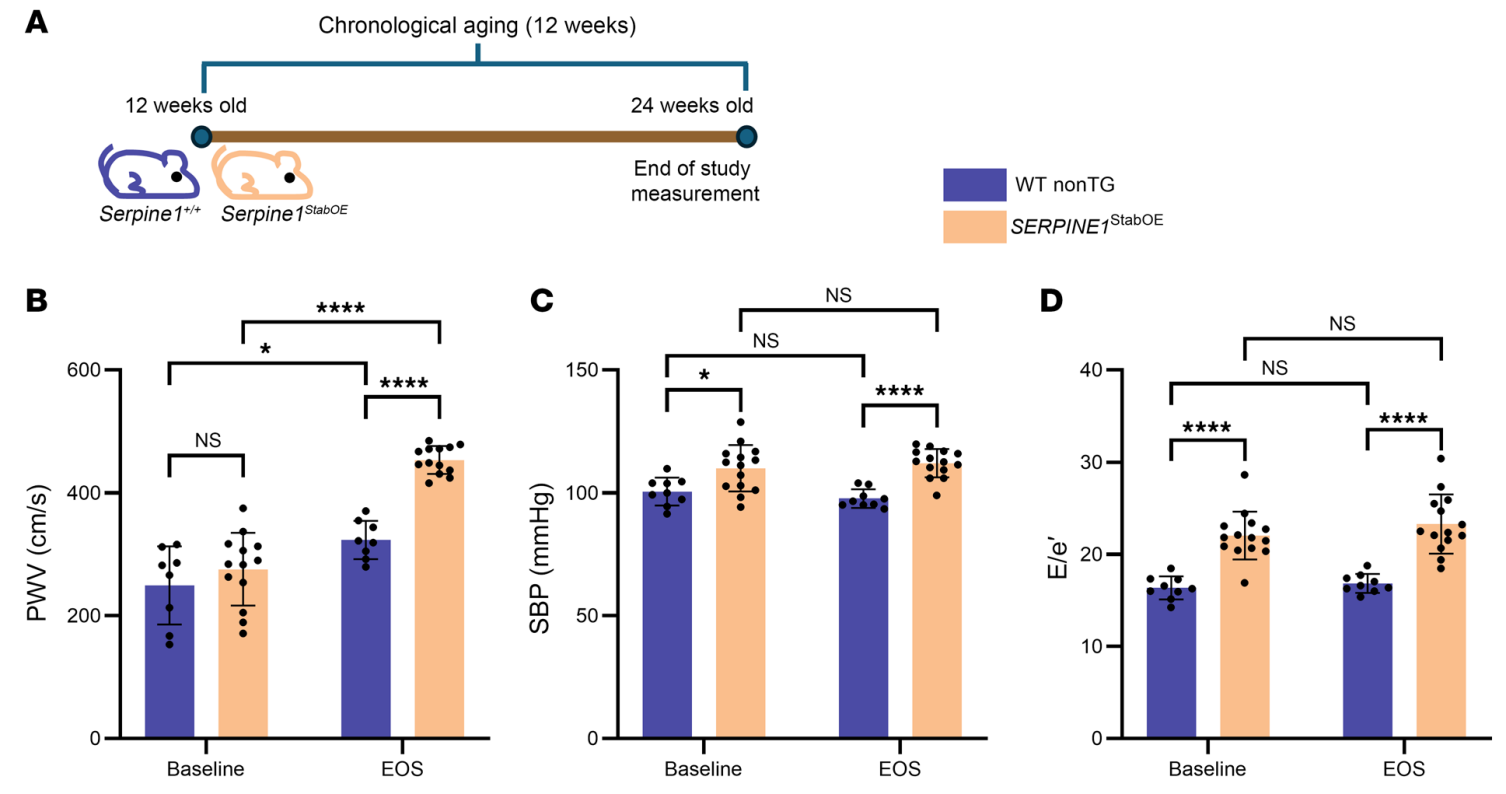
*SERPINE1*^StabOE^ mice exhibit elevated cardiovascular pathophysiology. (**A**) Schematic of the study: *n* = 9 WT nonTG mice (5 males and 4 females); *n* = 14 *SERPINE1^StabOE^* mice (5 males and 9 females) used for SBP measurements and *n* = 13 *SERPINE1^StabOE^* mice (4 males and 9 females) used for E/e′ and PWV measurements. (**B**) PWV (**C**) SBP, and (**D**) E/e′ for *SERPINE1^StabOE^* mice compared with littermate controls at 12 weeks of age (baseline) and at 24 weeks of age (EOS). *P < 0.05 and ****P < 0.0001, by 2-way ANOVA (**B**–**D**) to determine significant interactions between variables.

Given our results in mice with genetic reduction in PAI-1, we hypothesized that overexpression of PAI-1 would heighten the adverse increases in vascular physiology measures that we observed in l-NAME–treated mice. To test this hypothesis, we utilized a mouse model that expresses a stabilized form of human PAI-1 under the murine preproendothelin 1 promoter, maintained on a B6.D2 background (hereafter referred to as *SERPINE1^StabOE^* mice) (16). *SERPINE1^StabOE^* mice and their WT littermate controls underwent baseline physiological studies at 12 weeks of age and reassessment at 24 weeks of age (end of study) (Figure 3A). At baseline, *SERPINE1^StabOE^* mice showed elevated SBP and E/e′ compared with littermate controls (Figure 3, C and D), indicating that excess PAI-1 can increase these measures as early as 12 weeks of age. We reassessed the same mice after 12 weeks of aging alone (i.e., at 24 weeks of age) and found further increases in PWV, SBP, and E/e′ in *SERPINE1^StabOE^* mice compared with control mice (Figure 3, B–D). Stratifying measurements by biological sex confirmed consistent and statistically significant differences across all groups (Supplemental Figure 6). The physiologic observations suggest that excess PAI-1 expression is sufficient to drive an increase in aging-like pathophysiological changes by 12 weeks of age with progressive worsening in vascular stiffening or vascular tone at 24 weeks of age.

### Bulk RNA-Seq identifies Ccn1 as a differentially expressed gene in Serpine1^TA700/+^ aortas exposed to l-NAME.

**Figure 4:**
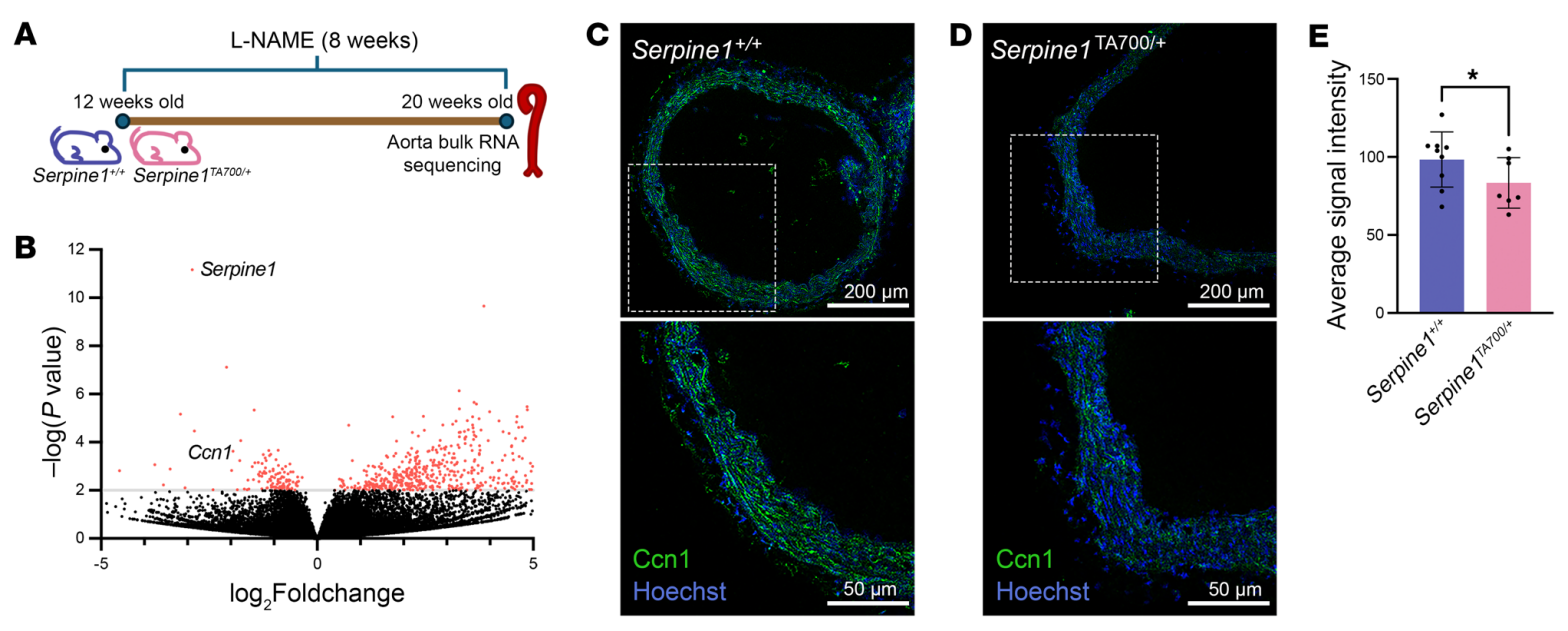
*Ccn1* transcript and protein expression is altered in aortas from *Serpine1^TA700/+^* mice. (**A**) Schematic of the study. (**B**) Differentially expressed genes in *Serpine1^TA700/+^* (*n* = 3 males and *n* = 3 females) compared with littermate controls (*n* = 3 males and *n* = 3 females) using bulk RNA-Seq of aortas. (**C** and **D**) Confocal images of immunofluorescent signals of CCN1 (green) and nuclei (blue) in aortas from *Serpine1*^+/+^ (**C**) and *Serpine1*^TA700/+^ (**D**) mice. (**E**) Quantification of aortic CCN1 signal intensity from 9 *Serpine1*^+/+^ mice (*n* = 4 males; *n* = 5 females) and 7 *Serpine1*^TA700/+^ mice (*n* = 4 males; *n* = 3 females) mice. *P < 0.05, by DESeq2 (**B**) and 1-tailed Mann-Whitney *U* test (**E**). Scale bars: 50 μm and 200 μm.

Building on our physiological findings, we investigated specific pathways that may mediate the protection observed in *Serpine1^TA700/+^* following l-NAME. We first performed bulk RNA-Seq on the aortas of *Serpine1^TA700/+^* and control littermates following 8 weeks of l-NAME (Figure 4A). *Serpine1* was among the most significantly reduced differentially expressed genes in *Serpine1^TA700/+^* aortas, consistent with the genetic reduction of *Serpine1* in this model. RNA-Seq also revealed significantly reduced expression of *Ccn1* in *Serpine1^TA700/+^* aortas compared with controls after 8 weeks of l-NAME treatment (Figure 4B). We then used immunofluorescence to visualize differences in CCN1 protein (Figure 4, C–E) and found that *Serpine1^TA700/+^* aortas had reduced CCN1 protein expression. These data highlight the effects of PAI-1 reduction on *Ccn1* expression and CCN1 protein levels.

### Aorta scRNA-Seq differences in Serpine1^TA700/+^ mice exposed to l-NAME.

**Figure 5:**
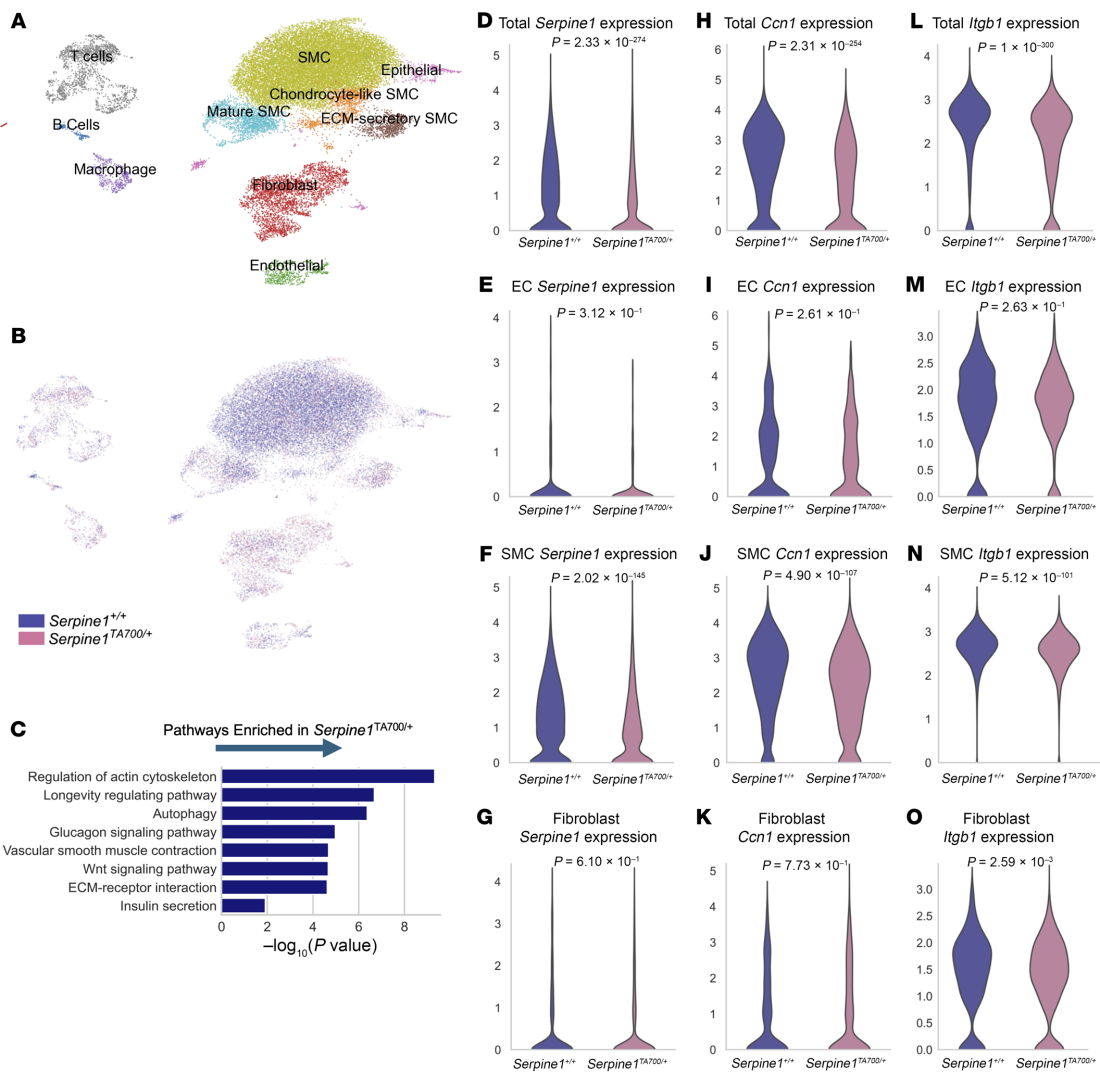
scRNA-Seq of aortas from *Serpine1^TA700/+^* and *Serpine1^+/+^* mice. (**A**) UMAP plot showing the clustering of all cells sequenced from aortas of 4 *Serpine1^TA700/+^* mice (*n* = 2 males and *n* = 2 females) and 6 *Serpine1^+/+^* mice (*n* = 3 males and *n* = 3 females). (**B**) UMAP plot showing the composition of sequenced cells from mice of each genotype. (**C**) Pathway enrichment analysis was done on genes overrepresented in total *Serpine1^TA700/+^* and *Serpine1^+/+^* cells using KEGG. (**D**–**O**) Violin plots showing log-normalized gene expression of *Serpine1*, *Ccn1*, and *Itgb1* in total cells, ECs, SMCs, and fibroblasts from mice of the 2 genotypes. The Mann-Whitney *U* test was used to test for significance in the differential expression of genes (**D**–**O**).

To gain deeper insights into the cellular mechanisms underlying the observed transcriptional changes, we performed scRNA-seq on independent aorta samples using the same study design as bulk RNA-Seq (Figure 4A). This approach allowed us to dissect the cellular composition and gene expression dynamics driving the vascular phenotype. We sequenced 29,535 cells from the aortas of 4 *Serpine1^TA700/+^* and 6 control mice (Figure 5A). The *Serpine1^TA700/+^* and control aortas yielded 12,001 and 17,534 cells, respectively (Figure 5B). More than 60% of the sequenced cells were identified as vascular smooth muscle cells (SMCs), while endothelial cells (ECs) accounted for just over 2% of the sequenced cells. According to earlier literature on SMCs (42, 43), we used the gene markers *Tnfrsf11b*, *Lgal3*, and *Acan* to identify chondrocyte-like SMCs that resemble chondrocytes (Supplemental Figure 7A). We also defined extracellular matrix–secretory (ECM-secretory) SMC as cells with moderate expression of contractile markers such as *Myh11* and *Mylk* (but low *Acta2* expression) (44), as well as high expression of ECM protein genes such as *Vcan* that have been associated with “young” ECM (45, 46) (Supplemental Figure 7B). Using Kyoto Encyclopedia of Genes and Genomes (KEGG) pathway enrichment, we found that cells from *Serpine1^TA700/+^* aortas were enriched for pathways critical for vascular aging such as longevity regulation, autophagy (47), and FoxO signaling (48) (Figure 5C).

When examining specific genes in our dataset, we observed that aortic *Serpine1* expression in *Serpine1*^TA700/+^ mice was lower when compared with control (Figure 5, D–G). We found the largest difference in *Serpine1* transcripts in SMC clusters, whereas fibroblasts and ECs exhibited little difference (Figure 5, D–G, and Supplemental Figure 7C). Consistent with our bulk RNA-Seq results, we found reduced aortic *Ccn1* expression in *Serpine1*^TA700/+^ cells compared with control cells (Figure 5, H–K). CCN1 protein (also known as CYR61) (49) has been shown to interact with integrin β1 (ITGβ1) to promote senescence (50–52), a hallmark of cardiovascular aging (53). Interestingly, our scRNA-Seq data analysis revealed reduced expression of the gene encoding ITGβ1, *Itgb1*, in *Serpine1^TA700/+^* aortas compared with controls (Figure 5, L–O). The greatest difference in *Ccn1* and *Itgb1* expression between the 2 genotypes was observed in SMC clusters (Figure 5, J and N, and Supplemental Figure 7, D and E). This finding suggests that reduced *Itgb1* expression in *Serpine1^TA700/+^* may attenuate ITGβ1 signaling, further limiting the prosenescent effects of CCN1 and contributing to vascular protection.

**Figure 6:**
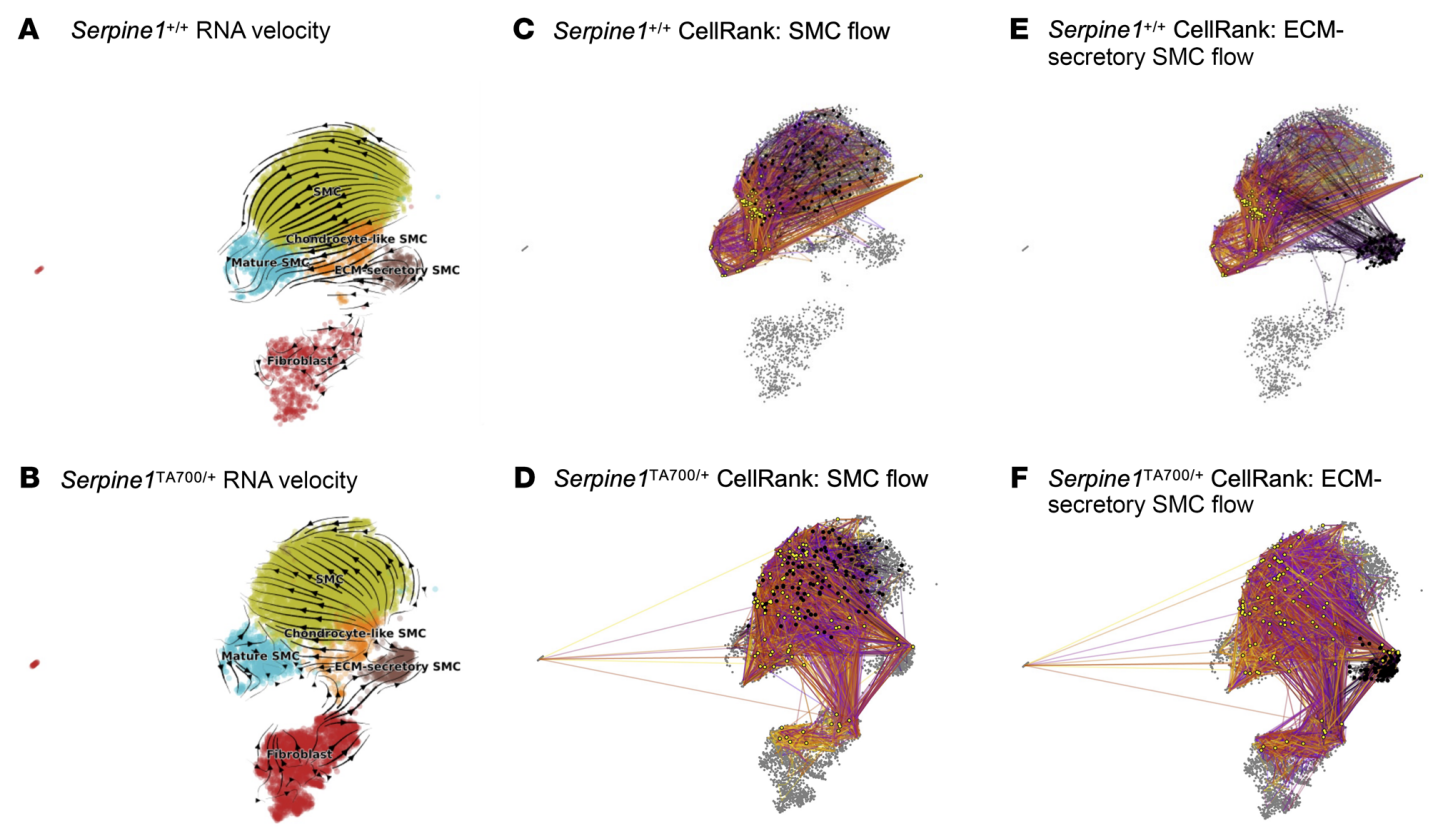
RNA velocity and CellRank analyses reveal altered cell-state dynamics in *Serpine1^TA700/+^* aortas. (**A** and **B**) Steady-state RNA velocity stream plots showing transcriptional dynamics within fibroblast and SMC lineages in *Serpine1^+/+^* (**A**) and *Serpine1^TA700/+^* (**B**) mouse aortas. Arrows indicate the inferred direction of cell-state transitions, revealing more restricted trajectories in controls and greater plasticity in *Serpine1^TA700/+^* mice. (**C**–**F**) CellRank fate-mapping visualizations indicating transitions from SMCs (**C** and **D**) and ECM-secretory SMCs (**E** and **F**). Initial states are shown in black, terminal states in yellow, and directed edges represent cell-fate probabilities from each starting point. Darker arrows represent higher transition probabilities in *Serpine1^+/+^* (**C** and **E**) and *Serpine1^TA700/+^* (**D** and **F**) aortas.

To further investigate the dynamics of cell state transitions within the SMC lineage, we performed steady-state RNA velocity (54) and CellRank (55) analyses on the scRNA-Seq data (Figure 6). In *Serpine1^+/+^* aortas, RNA velocity vectors followed consistent trajectories within SMC subclusters toward mature SMCs, suggesting limited transition to alternative fates (Figure 6A). In contrast, *Serpine1^TA700/+^* aortas displayed more diverse velocity patterns, suggesting increased transcriptional plasticity (Figure 6B). Notably, SMCs in *Serpine1^TA700/+^* mice exhibited trajectories toward ECM-secretory SMCs and fibroblasts, whereas SMCs and fibroblasts in control aortas remained confined to their cluster (Figure 6, C and D). CellRank further supported these findings by predicting that ECM-secretory SMCs in *Serpine1^TA700/+^* aortas could transition back to other SMC states (Figure 6F). In *Serpine1^+/+^* aortas, these ECM-secretory SMCs demonstrated an enhanced potential to transition to mature SMCs (Figure 6E). These findings suggest that PAI-1 reduction enhances the flexibility of SMC cell states, potentially promoting adaptive remodeling and limiting the terminal differentiation programs associated with vascular aging and pathology.

### Pharmacological PAI-1 inhibition reverses l-NAME–induced increase in PWV.

**Figure 7:**
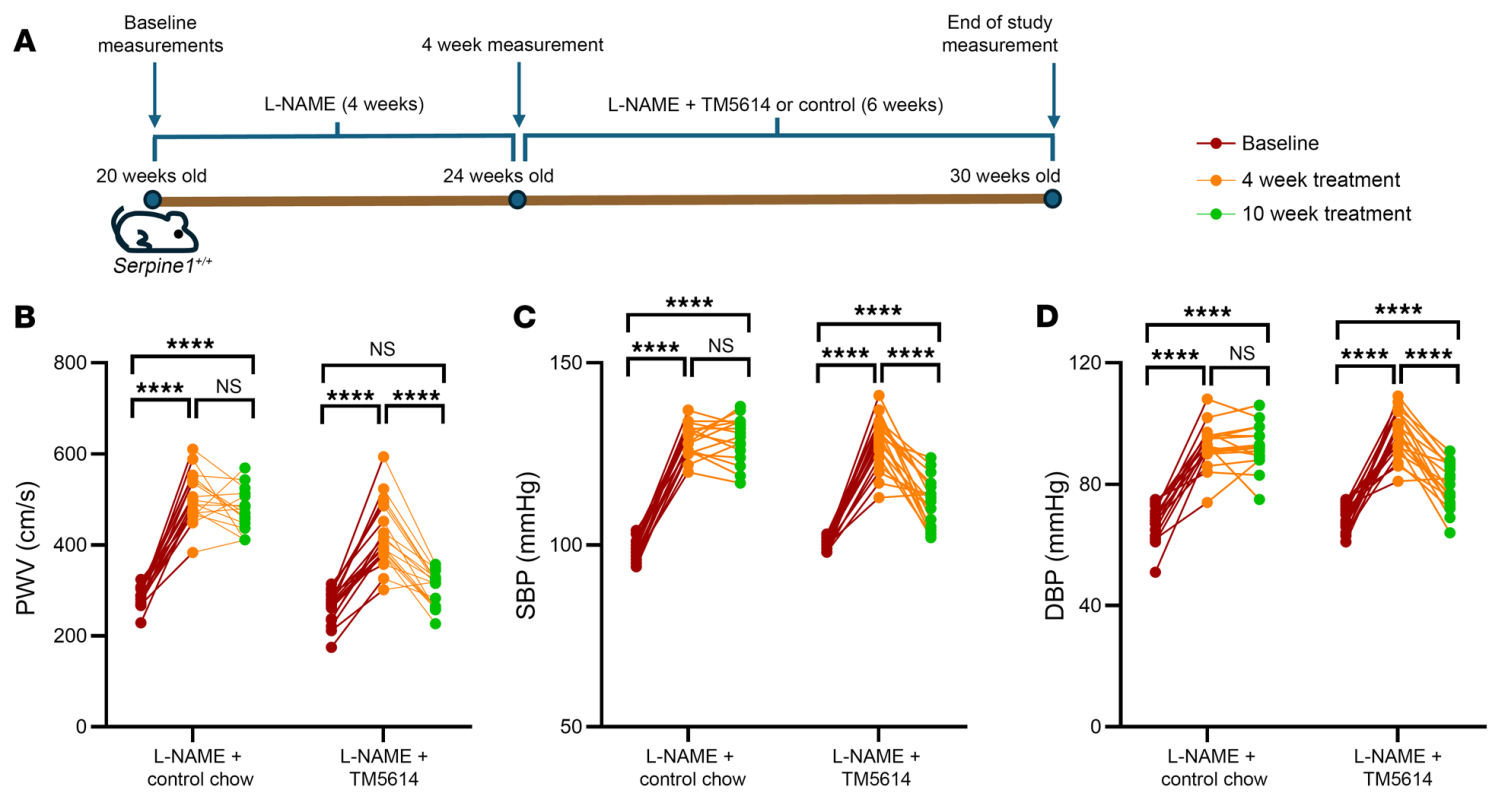
TM5614 administration reverses the l-NAME–induced increase in BP and PWV. (**A**) Schematic of the study. (**B**) PWV, (**C**) SBP, and (**D**) DBP of mice receiving control chow (left) or TM5614 (right) at baseline, 4 weeks, and at the end of the study. Mice receiving control chow: *n* = 7 males and *n* = 9 females; mice receiving TM5614: *n* = 9 males and *n* = 10 females. ****P < 0.0001, by ordinary 1-way ANOVA with Tukey’s correction for multiple comparisons (**B**–**D**).

Last, we investigated whether pharmacological PAI-1 inhibition can attenuate l-NAME–induced vascular pathophysiology. We administered l-NAME to 20-week-old *Serpine1^+/+^* mice for 4 weeks, followed by 6 weeks of combined l-NAME and PAI-1 inhibitor TM5614 treatment or control chow; during these treatments, we monitored changes in PWV at baseline, after l-NAME exposure, and after the combined treatment (Figure 7A). Consistent with prior results, l-NAME treatment led to significant increases in PWV, SBP, and diastolic blood pressue (DBP) (Figure 7, B–D). Remarkably, TM5614 administered at week 4 restored PWV to baseline levels at 10 weeks despite ongoing l-NAME treatment (Figure 7B). Additionally, we found that the subsequent TM5614 cotreatment significantly reduced the l-NAME–induced increase in SBP (Figure 7C) and DBP (Figure 7D). In contrast, the mice given control chow exhibited high PWV, SBP, and DBP measurements that were comparable to those taken at 4 weeks. Stratification of the measurements by biological sex confirmed consistent and statistically significant differences across all groups (Supplemental Figure 8). These findings imply that PAI-1 inhibition can decrease and even reverse preexisting aging-related cardiovascular pathophysiology.

## Discussion

We previously demonstrated that a rare LOF variant in the human *SERPINE1* gene is associated with protection against various aspects of biological aging (25). This study extends those findings by characterizing additional members of the Amish community with the mutation and providing additional evidence for the “heterozygous advantage” model. In this model, low-to-moderate levels of PAI-1 protect organisms from aging, while complete PAI-1 deficiency leads to cardiac fibrosis (56, 57), and excess PAI-1 drives accelerated aging (5, 7). Individuals who are homozygous for the *SERPINE1* LOF mutation develop mesocardial and subepicardial cardiac fibrosis through TGF-β signaling (56, 57). Similarly, *Serpine1^–/–^* mice develop age-dependent infiltrative cardiac fibrosis (58, 59). However, detailed protective mechanisms of the heterozygous advantage remain challenging to define in human populations. To address this barrier and further investigate physiologic and molecular roles for PAI-1 in vascular aging, we generated a mouse model (*Serpine1^TA700/+^*) that faithfully recapitulated the human mutation. Here, we show that *Serpine1^TA700/+^* mice were protected from l-NAME–induced cardiovascular pathophysiology, which indicates that elevated PAI-1 activity is necessary for vascular aging. While the l-NAME model is not a perfect representation of vascular aging, and its effects on neuronal NOS and central regulation are not fully accounted for, we show that the resulting phenotype of elevated PWV closely resembled that observed during natural aging (Figure 1F). We also used transgenic overexpression of PAI-1 (*SERPINE1^StabOE^*) to show that a significant excess of PAI-1 was sufficient to drive premature vascular aging. Last, and most clinically relevant, we found that the oral PAI-1 inhibitor TM5614 was able to reverse l-NAME–induced cardiovascular pathophysiology.

These findings provide a substantial contribution to the already compelling evidence that plasma PAI-1 is an important clinical predictor of vascular stiffness, hypertension, and diastolic dysfunction, especially in individuals with obesity and diabetes. Given the rarity of LOF mutations in *SERPINE1*, it is likely that the 43% of the world’s population with visceral obesity (2.5 billion people over the age of 18 years) (60) have a durable and consequential systemic excess of PAI-1, as BMI is the dominant determinant of plasma PAI-1 levels (61). Understanding the molecular mechanisms underlying PAI-1 reduction will rapidly translate into reducing age-related CVD for billions of people worldwide.

At the molecular level, we identified *Ccn1* and *Itgb1* as candidate genes potentially mediating the downstream effects of PAI-1. These observations raise important questions about the mechanisms through which PAI-1 influences the expression of these genes. Prior studies have implicated *Ccn1* in pathological vascular remodeling (62), fibrosis (63), and atherosclerosis (64). Moreover, CCN1 (50) utilizes integrin β1 to induce downstream signaling, and, interestingly, the gene encoding for ITGβ1 (*Itgb1*) was also downregulated in *Serpine1^TA700/+^* mice. We hypothesize that suppression of the *Ccn1* and *Itgb1* axis by PAI-1 reduction may serve as a protective mechanism. The elevated expression of these genes in the presence of high PAI-1 levels underscores the need for further investigation into how PAI-1 modulates the transcription and downstream signaling pathways of these genes. Although ECs lining the vasculature have been considered primary contributors of PAI-1 to the bloodstream in pathological conditions (65, 66), scRNA-Seq showed that *Serpine1* expression was more prominent in SMCs of the aorta than in ECs; our results are in keeping with a recent study of calcineurin-mediated hypertension and abdominal aortic aneurysm in mice (67).

Additionally, steady-state RNA velocity analysis suggested that SMCs from *Serpine1*^TA700/+^ mice were more likely to adopt an ECM-secretory SMC phenotype with low-to-moderate expression of contractile genes and high expression of ECM genes (Figure 6). CellRank analysis further suggested that SMCs from *Serpine1^+/+^* mice adopted a mature SMC phenotype with minimal plasticity, whereas SMCs from *Serpine1^TA700/+^* mice displayed higher plasticity and a greater likelihood to adopt the ECM-secretory phenotype after l-NAME exposure. ECM genes with high levels of expression in the ECM-secretory SMC population include *Lama2* and *Vcan*. These genes have been implicated in the maintenance of healthy, “young” ECM in other tissues. Mutations in *LAMA2* cause forms of muscular dystrophy due to weak interstitial connections between striated muscle cells (68). In neonatal myocardium, *Vcan* was identified as a proproliferative proteoglycan supporting cardiomyocyte proliferation (45, 46). Additional studies are needed to further investigate this population of SMCs, their role in maintaining a “young” ECM that maintains pliant vasculature, and how excess PAI-1 promotes the transition toward SMC populations that reduce vascular compliance.

Remarkably, TM5614 administration ameliorated the cardiovascular pathophysiology observed in WT mice subjected to l-NAME. These results align with earlier studies demonstrating the therapeutic potential of PAI-1 inhibitors in mitigating metabolic dysfunction (19, 23, 69, 70), fibrosis (21), hypertension (67), and muscle atrophy (71). Recently, clinical trials have involved the testing of TM5614 in patients with chronic myelogenous leukemia (72), metastatic melanoma (73), non–small cell lung cancer (74), and angiosarcoma (75). Previous studies in humans carrying the *SERPINE1*^TA700/+^ mutation provide compelling evidence that lifelong partial PAI-1 deficiency is both well tolerated and protective. This human-based insight anchors the translational potential of PAI-1 inhibition into effective clinical strategies. Together with ongoing clinical trials exploring TM5614 in cancer, our findings demonstrate that TM5614 can also enhance organismal fitness, especially in delaying, or even reversing, cardiovascular aging.

## Methods

### Sex as a biological variable

Our study examined both male and female individuals and animal models. For humans and animals, data were analyzed with the sexes aggregated as well as separately.

### Human studies

The population comprises individuals from an Old Order Amish population in Berne, Indiana (USA) that harbor the c.699_700dupTA frameshift mutation in *SERPINE1* that was previously described (25); this cross-sectional observational study included additional members (33 *SERPINE1^TA700/+^* and 33 *SERPINE1^+/+^*) that were not part of the original 2017 study. Participants were genotyped using PCR amplification specific for the c.699_700dupTA frameshift. The study participants underwent PWV measurement by an experienced sonographer who was blind to participant genotype. PWV was measured noninvasively using the SphygmoCor XCEL Vascular Biometric Monitor (AtCor Medical, Australia) according to the manufacturer’s standard protocol. Participants were placed in a supine position, and measurements were obtained following a 10-minute rest period. Carotid-femoral PWV was calculated using applanation tonometry and oscillometric cuff data. To age and sex match the values from *SERPINE1^TA700/+^* participants, PWV values from the same age and sex *SERPINE1^+/+^* participants were randomly chosen to match participants with the *SERPINE1^TA700/+^* mutation.

### Animal models

#### Serpine1^TA700/+^ mice.

To generate an equivalent mouse model harboring the human *SERPINE1^c.699_700dupTA^* mutation on the C57BL6/J genetic background, a CRISPR/Cas9 gene-editing approach was used. sgRNAs (5′-CAGTGAGTCCAAATATCCCC-3′, 5′-GTCTCATAACTACTGACCCT-3′, and 5′-TGTCTCATAACTACTGACCC-3′) and a donor DNA template (5′-CTTTCTTAGAGGCCAGCACCCACCAGCGCCTCTTCCACAAGTCTGATGGCAGCACNGTCTCTGTGCCCATGATGGCTCAGAGCAACAAGTTCAACTACATA GTGAGTCCAAATATCCCCAGGTTCCATGTCTCATAACTACTGACCCTGGGCAACATTCACAGTGTCCCTCTCAGAGGGAGGATGGTGGCAGAGGAGGTG-3′) were designed to introduce a global TA dinucleotide insertion at the end of exon 4 of the mouse *Serpine1* gene. This insertion created a premature stop codon, resulting in a truncated mPAI-1 289 amino acid polypeptide, and concurrently abolished the BfmI (SfcI) restriction site at this location. The CRISPR/Cas9 reagents and repair template were electroporated into C57BL6/J zygotes at the Transgenic and Targeted Mutagenesis Laboratory of Northwestern University. Following electroporation, the zygotes were implanted into pseudopregnant female mice, and 20 F0 progeny were obtained. Genomic DNA was extracted from tail biopsies of F0 mice and analyzed to confirm the presence of the desired mutation. The presence of the TA dinucleotide insertion was confirmed by Sanger sequencing in 3 of the 20 F0 mice, which were found to be heterozygous. PCR amplification followed by SfcI digestion was performed as a genotyping strategy to screen for the mutation, as the TA insertion disrupted the SfcI recognition site at the end of exon 4. The resulting PCR amplicons from mutant alleles were resistant to SfcI digestion, whereas WT alleles were cleaved. These heterozygous F0 mice were used as the founders for subsequent breeding and maintenance on the C57BL6/J background to establish a colony for this work.

#### SERPINE1^StabOE^ mice.

For PAI-1–transgenic mice, which were previously described (16), the murine preproendothelin-1 promoter was used to drive expression of a cDNA coding for the functionally stable variant of human PAI-1. These mice were maintained on the B6.D2 background.

#### Serpine1-GFP–transgenic reporter mice.

We previously reported the generation of a transgenic mouse line, 3kPAI-1-EGFP, that overexpresses enhanced GFP under the control of the 3 kb proximal promoter of the human PAI-1 gene promoter (76). This mouse line was maintained on the B6.D2 background and used for the experiment involving 10 weeks of l-NAME treatment with concomitant TM5614 versus vehicle during the last 6 weeks of the experiment (Figure 6). This mouse line has no gene mutation at the *Serpine1* locus; all mice in this line are *Serpine1*^+/+^.

### l-NAME administration

l-NAME was used to inhibit eNOS and recapitulate the vascular stiffening associated with increasing age. At 12 weeks of age, *Serpine1^TA700/+^* and *Serpine1^+/+^* mice were given l-NAME (Thermo Fisher Scientific, H63666.14) in drinking water (1 mg/mL) for 8 weeks. Cardiovascular physiology (described later) was performed at baseline and after 8 weeks of l-NAME administration. For transcriptomics characterization, aortas were harvested after 8 weeks of l-NAME administration (described below).

### TM5614 administration

l-NAME treatment in conjunction with the PAI-1 inhibitor TM5614 (Renascience) was used to capture the effects of pharmacological PAI-1 inhibition on vascular stiffening. Twenty-week-old *Serpine1*-GFP–transgenic reporter mice underwent baseline cardiovascular characterization, received l-NAME (1 mg/mL) for 4 weeks, and cardiovascular characterization was repeated. For the following 6 weeks, the mice continued 1 mg/mL l-NAME; half of the mice received the oral PAI-1 inhibitor TM5614 (20 mg/kg/day) mixed in the standard chow, while the other half received control chow. BP and PWV measurements were performed a third time, and animals were euthanized for aorta collection.

### Cardiovascular physiology

All measurements described below were performed in a manner blinded to the genotype and/or treatment.

### Echocardiography

Diastolic parameter E/e′ was measured using the VEVO3100 ultrasound machine (Fujifilm VisualSonics). Mice were placed under anesthesia with isoflurane and positioned on a heating pad in a supine position, with anesthesia titrated to maintain heart rates above 500 for systolic and approximately 500 for diastolic measurements. Body temperature was controlled between 37°C using a heating lamp. The high-frequency ultrasound probe MX550D (25–55 MHz) (Fujifilm VisualSonics) was used to capture 4-chamber and left ventricular parasternal long and short-axis views of the heart. The data were subsequently analyzed for systolic and diastolic function measurements, as well as myocardial strain measurements using VEVO Lab software (Fujifilm VisualSonics).

### BP measurements

Systolic BPs were measured using a noninvasive tail-cuff device (CODA High-Throughput System from Kent Scientific). Mice were placed conscious in conical holders for 15 minutes to acclimatize, and BP measurements were taken over 40 cycles.

### PWV measurements

PWV was measured in mice using the MX550D ultrasound probe on the VEVO3100 ultrasound machine (Fujifilm VisualSonics). Mice were placed under anesthesia with isoflurane and positioned in a supine position on a heating pad, with anesthesia titrated to maintain a heart rate above 500. Two pulse-wave Doppler images were obtained from the aorta, one proximal and one distal. B-Mode images were captured and analyzed using VEVO Lab software (Fujifilm VisualSonics), with the distance measured between the proximal and distal pulse waves, and the time from the QRS peak to the pulse wave in the aorta was measured at these 2 locations. The software calculated the PWV using the distance and time between the 2 set points.

### Immunofluorescence

Aortas were harvested from mice and embedded in OCT. Cryosectioning was used to obtain 10 μm thick sections. The slides were permeabilized in 0.1% Triton-X 100 for 3 minutes, blocked in 0.2% BSA PBS for 1 hour, incubated with a polyclonal CCN1 antibody raised in rabbit (Proteintech, 26689-1-AP) at 4°C for 3 hours, washed 3 times with PBS, incubated with a goat anti–rabbit IgG (H+L) secondary antibody conjugated with Alexa Fluor 488 (Thermo Fisher Scientific, A-11008), washed 3 times with PBS, dyed with Hoechst 33342 (Thermo Fisher Scientific, H3570), washed 3 times, and then mounted with Vectashield (Vector Laboratories, H-1000). Slides were kept in a refrigerator until imaging.

Slides were then imaged using a Zeiss LSM980 microscope. Low-power images shown in Figure 4, C and D were taken using a Plan Apochromat ×10/0.45 NA objective with the 488 laser at 1.2% power and identical contrast for all images. High-power images were taken using a Plan Apochromat ×63/1.3 NA high-resolution objective (Zeiss Group). All images used in quantification (reported in Figure 4E) were taken using the ×63 objective with the 488 nm laser at 0.08% power and identical contrast for all images. The intensity of the fluorescence signal in stained slides was analyzed by a team member blinded to the genotype using ImageJ software (NIH). Briefly, images were converted to grayscale for the green and blue color channels, and the intensity value for every pixel was determined by the function “analyze histogram” of ImageJ. The aggregate intensity of all pixels was divided by the number of pixels with a value over threshold of 100, which was determined experimentally on the basis of the background fluorescence, and the final value is presented as “mean intensity” for each slide.

### ELISA

Circulating PAI-1 antigen levels in *Serpine1^TA700/+^*, *Serpine1^TA700/TA700^*, and *Serpine1^+/+^* mice were detected using a commercial ELISA kit for mouse total PAI-1 antigen (Innovative Research, IMPAI1KTT) according to the manufacturer’s protocol.

### Transcriptomics

#### Bulk RNA-Seq.

Following l-NAME treatment, *Serpine1^TA700/+^* and *Serpine1^+/+^* mice were euthanized in a CO_2_ chamber for 2 minutes. The aortas were isolated and underwent mild cleaning of the periaortic fat and excess tissue. RNA was isolated using the QIAGEN RNeasy Mini Kit (QIAGEN) according to the manufacturer’s protocol. RNA samples were sent to Novogene America (Novogene America) for next-generation mRNA-Seq. The aligned reads were analyzed using Python 3.9 (Python Software Foundation).

#### scRNA-Seq.

Single-cell analysis of l-NAME–treated *Serpine1^TA700/+^* and *Serpine1^+/+^* aortas was performed by adapting a previously described digestion protocol (43). A working enzyme solution was prepared on ice by combining Liberase (Roche, 05401127001) at 2 units/mL final concentration, elastase (Worthington, LS002279) at 2 units/mL final concentration, and DNase I at 60 units/mL (Roche, 10104159001) in HBSS with Ca^2+^ and Mg^2+^ (Gibco, Thermo Fisher Scientific, 14025092).

After euthanasia in a CO_2_ chamber for 2 minutes, mice were perfused with 10 mL HBSS (Gibco, Thermo Fisher Scientific, 14025092) via left ventricular puncture, and the aorta was harvested. The aortas underwent extensive cleaning of periaortic fat and excess tissue while submerged in MACS Separation Buffer (Miltenyi Biotec, 130-091-221). The aorta was split open and minced using iris scissors, transferred to 1 mL enzyme solution as described above, and incubated at 37°C for 45 minutes while shaking at 300 rpm. The digestion reaction was stopped by adding 5 mL 2% FBS (Gibco, Thermo Fisher Scientific, A38401-01) in HBSS (Gibco, Thermo Fisher Scientific, 14025092). The cell suspension was passed through a 70 μm strainer into a 50 mL conical tube, and residual liquid was collected by centrifugation at 300*g* for 10 minutes. Isolated cells were transferred into a microcentrifuge tube and centrifuged at 300*g* for 5 minutes. All but 100 μL supernatant was removed, and cells were resuspended in the remaining 100 μL volume. Cell viability was determined with the Nexcelom Cellometer cell counter (Revvity), with an average of 86.3% viability and a range of 71%–94%.

Cell suspensions were submitted to the Integrative Genomics Core at the Robert H. Lurie Comprehensive Cancer Center of Northwestern University for partitioning and generation of transcriptomic libraries. Single-cell mRNA libraries were built using the Chromium Next GEM Single Cell 3′ Library Construction V4 Kit. Libraries were sequenced using a high-output P4 flow cell (XLEAP Chemistry) on an Illumina NextSeq 2000 sequencer, which generates up to 1.8 billion reads per run. Sequencing data were aligned to the mouse reference using the Cell Ranger 3.0.2 pipeline (10X Genomics). The data were analyzed using Python 3.9 (Python Software Foundation).

### Bioinformatics

#### scRNA-Seq preprocessing and analysis.

scRNA-Seq data from 32,354 cells were preprocessed to remove low-quality cells and doublets. Cells with greater than 8% mitochondrial gene expression and outliers based on total counts, detected genes, and top 20 gene expression were excluded. Doublets were identified using a doublet detection classifier python package (77) and removed. After filtering, data from 29,535 cells were used for further processing. Data were then normalized and integrated using scanpy (1.10.0) and harmony (0.0.10), respectively.

Cell clustering was performed using the scanpy Leiden algorithm (resolution = 0.5) on adjusted principal components derived from harmony. Uniform manifold approximation and projection (UMAP) was used to visualize the latent representation of the scRNA-Seq data. Data were normalized, log transformed, and visualized using scanpy UMAP. Differential gene expression analysis identified marker genes, which were used for manual cell-type annotation. UMAP was used to visualize the latent representation of the scRNA-Seq data.

After integration, the Wilcoxon rank-sum test (implemented in scanpy) in the “rank gene groups” function was used to compare gene groups and identify chondrocyte-like SMCs and ECM-secretory SMCs. Cell types were assigned using marker gene expression in clusters such as chondrocyte-associated markers (*Lgals3*, *Acan*, *Tnfrsf11b*) and fibroblast-associated markers (*Adamtsl1*, *Lama2*, *Fbln1*, *Vcan*). Gene expression was then used to distinguish chondrocyte-like SMCs and ECM-secretory SMCs, respectively.

#### Differential gene expression and pathway enrichment analysis.

Differential gene expression analysis was performed using the Wilcoxon rank-sum test on normalized counts to identify genes that were significantly upregulated or downregulated between genotypes. Significantly differentially expressed genes (*P* < 0.05, adjusted for multiple comparisons) were classified as upregulated or downregulated on the basis of their log-fold change values. Violin plots were used to visualize differentially expressed genes based on log-normalized expression values. The Mann-Whitney *U* test was then applied to compare the expression distributions of the gene between the 2 genotypes. KEGG pathway enrichment analysis was then conducted using gseapy’s “enrichr” function to determine biological pathways enriched among the upregulated and downregulated genes.

### RNA velocity analysis

scRNA-Seq data were processed as described above. For RNA velocity analysis, the following 3 steps were performed. First, spliced and unspliced transcripts were quantified using Velocyto (version 0.17.17) to create gene expression count matrices. Second, we subsetted by genotype and used scVelo (version 0.3.3) to log-normalize count matrices, and then a dynamical model was run to generate a UMAP representation with stream trajectories. Third, cellrank2 (version 2.0.7) was used to simulate random walks starting in the SMC cluster for both genotypes separately. Stream trajectories in the *Serpine1^TA700/+^* genotype indicated a “backflow” from the SMC cluster to the ECM-secretory SMC cluster, but this backflow was not present in WT mice (Figure 6, A and B). To confirm this finding, cellrank2 random walks projection was performed with the SMC cluster set as starting point (Figure 6, C and D) and the ECM-secretory SMC cluster set as a starting point (Figure 6, E and F). Cellrank2 revealed that in *Serpine1^+/+^* mice, the SMCs transitioned toward mature SMCs; in *Serpine1^TA700/+^* mice, the SMCs transitioned toward ECM-secretory SMCs and fibroblasts (Figure 6, C–F), indicating altered terminal states of SMCs between the 2 genotypes.

### Statistics

The number of samples and the specific statistical parameters used for each experiment are included in the figure legends. To assess the effect of genotype on PWV, a multiple linear regression model was performed with PWV as the dependent variable and genotype, age, and sex and included as covariates. Two simple linear regressions were also performed on each genotype using PWV as the dependent variable and age as the independent variable, in which analysis of covariance determined significance between the slope and elevation of the 2 separate regressions. Statistical testing was done using GraphPad Prism 10.3 (GraphPad Software). The threshold for statistical significance was set at a *P* value of less than 0.05. For all graphs with error bars, data are shown as mean with standard deviation, with the exception of Figure 1D, which shows IQR with minimum/maximum.

### Study approval

Northwestern University’s IRB approved the study protocol, and all participants provided written informed consent. All animal studies were approved by the Northwestern University IACUC, and all characterizations followed the guidelines set by Northwestern University.

### Data availability

All RNA-Seq data are available through the Gene Expression Omnibus (GEO) database (GEO GSE297835, aorta sequencing). The Supporting Data Values file includes all individual data points.

## Author contributions

LDW and DEV designed experiments, supervised the research, and wrote the manuscript. A. Khoddam, A. Kalousdian, and LDW performed experiments, analyzed data, and wrote the manuscript. ME, SS, AD, EJL, BWZ, BD, BSC, CMC, and HAV performed experiments and edited the manuscript. BWZ and BB analyzed data. AS analyzed data and edited the manuscript. TM provided materials and edited the manuscript. A. Khoddam organized and wrote the majority of the manuscript, and for this reason he is listed first among the 2 co–first authors.

## Funding support

This work is the result of NIH funding, in whole or in part, and is subject to the NIH Public Access Policy. Through acceptance of this federal funding, the NIH has been given a right to make the work publicly available in PubMed Central.

- NIH grants R35HL171553 (to DEV) and R01AI170938 (to CMC).
- AHA Predoctoral Fellowship (25PRE1356859, to A. Khoddam).
- National Cancer Institute (NCI), NIH (grant CCSG P30 CA060553, in support of transcriptomics and data analysis performed by Northwestern University’s Integrative Genomics branch of the Metabolomics Core Facility at Robert H. Lurie Comprehensive Cancer Center).

## Supplementary Material

Supplemental data

Supporting data values

## Figures and Tables

**Table 1:**
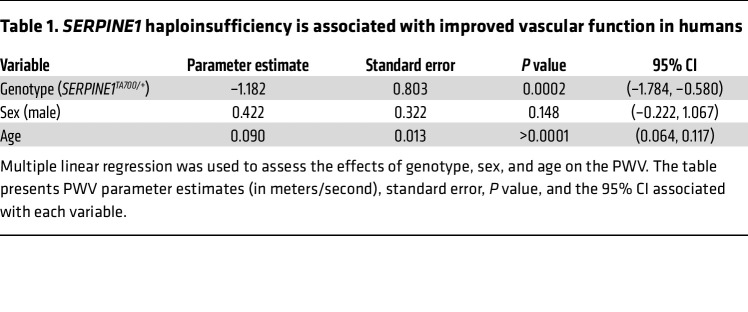
*SERPINE1* haploinsufficiency is associated with improved vascular function in humans

## Version 1 09/30/2025

In-Press Preview

## Version 2 12/01/2025

Electronic publication
