# Supplementary material for: Plasminogen activator inhibitor 1 promotes aortic aging–like pathophysiology in humans and mice
Source: J Clin Invest. 2025 Sep 30;135(23):e196714. doi: 10.1172/JCI196714 (PMC12646655; doi:10.1172/JCI196714)
Supplement: Supplemental data [file jci-135-196714-s337.pdf]

SUPPLEMENTAL DATA

Supplemental Figure 1.

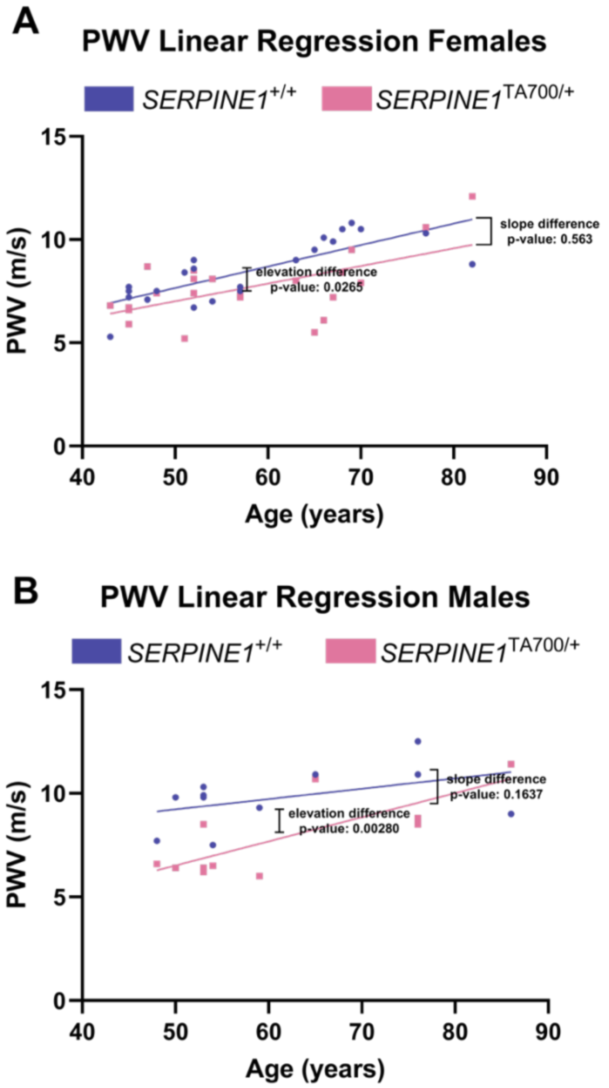

Supplemental Figure 1. The reduction in vascular stiffness associated with *SERPINE1*$^{TA700/+}$ genotype in humans is consistent across biological sex. Scatterplot of PWV as a function of age in age and sex matched *SERPINE1*$^{TA700/+}$ and *SERPINE1*$^{+/+}$ in (**A**) females (n= 36 per genotype) and (**B**) males (n=13 per genotype). Aggregated human PWV values in (**C**) females (n= 36 per genotype) and (**D**) males (n=13 per genotype). To determine significance, analysis of covariance was used in (**A**) and (**B**) and two-tailed unpaired t-test was used in (**C**) and (**D**). *, $p < 0.05$.

Supplemental Figure 2.

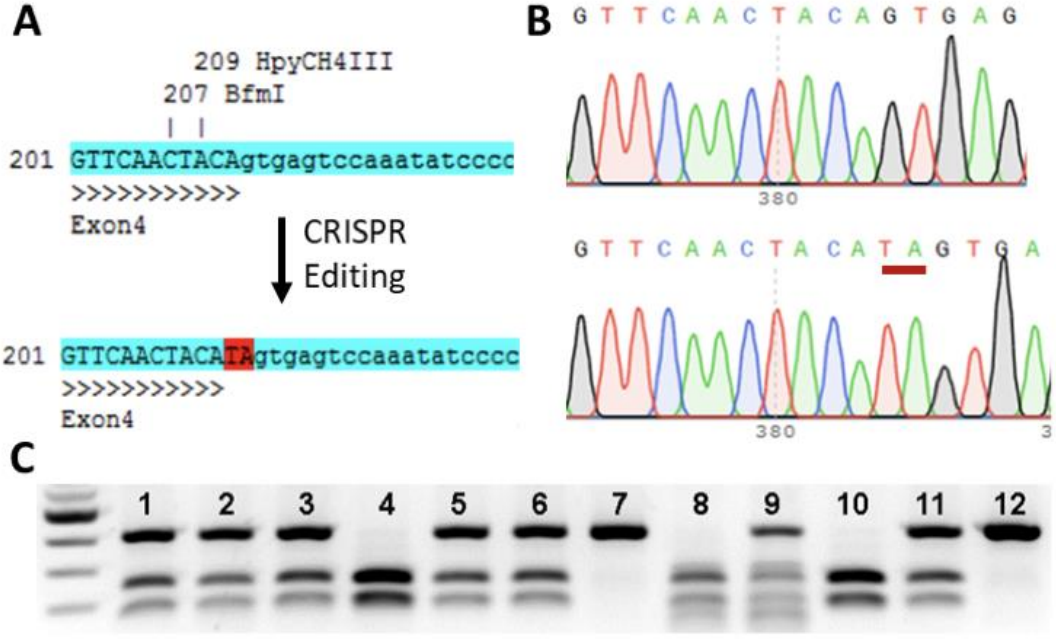

Supplemental Figure 2. Generation of *Serpine1*$^{TA700/+}$ mice. (**A**) sequence of the wildtype mouse Serpine1 gene (top) and the mutated gene (bottom) that harbor a TA dinucleotide insertion (red highlight). (**B**) Sanger sequencing of the wildtype mouse Serpine1 gene (top) and the mutated gene (bottom). (**C**) An example of genotyping of the novel mouse line. Since the dinucleotide insertion abrogates the BfmI digestion enzyme recognition site, *Serpine1*$^{TA700/TA700}$ mice have an undigested band (lanes 7 &12) while wildtype mice have only digested bands (lanes 4 & 10). *Serpine1*$^{TA700/+}$ mice have a combination of all three bands (lanes 1, 2, 3, 5, 6, 9, and 11).

Supplemental Figure 3.

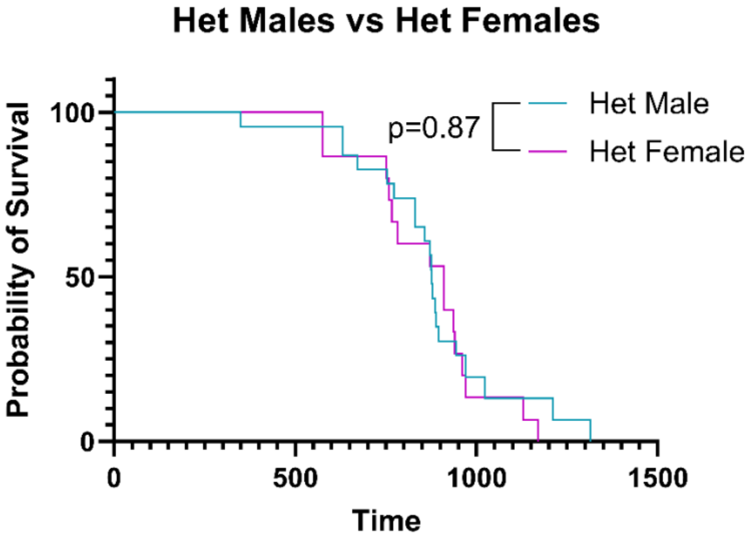

Supplemental Figure 3. Survival of *Serpine1*$^{TA700/+}$ (Het) males and females. Comparison of Het males and Het females. Log Rank (Mantel-Cox) test was used to test for significance. Number of biological replicates is reported in main Figure 1E.

Supplemental Figure 4.

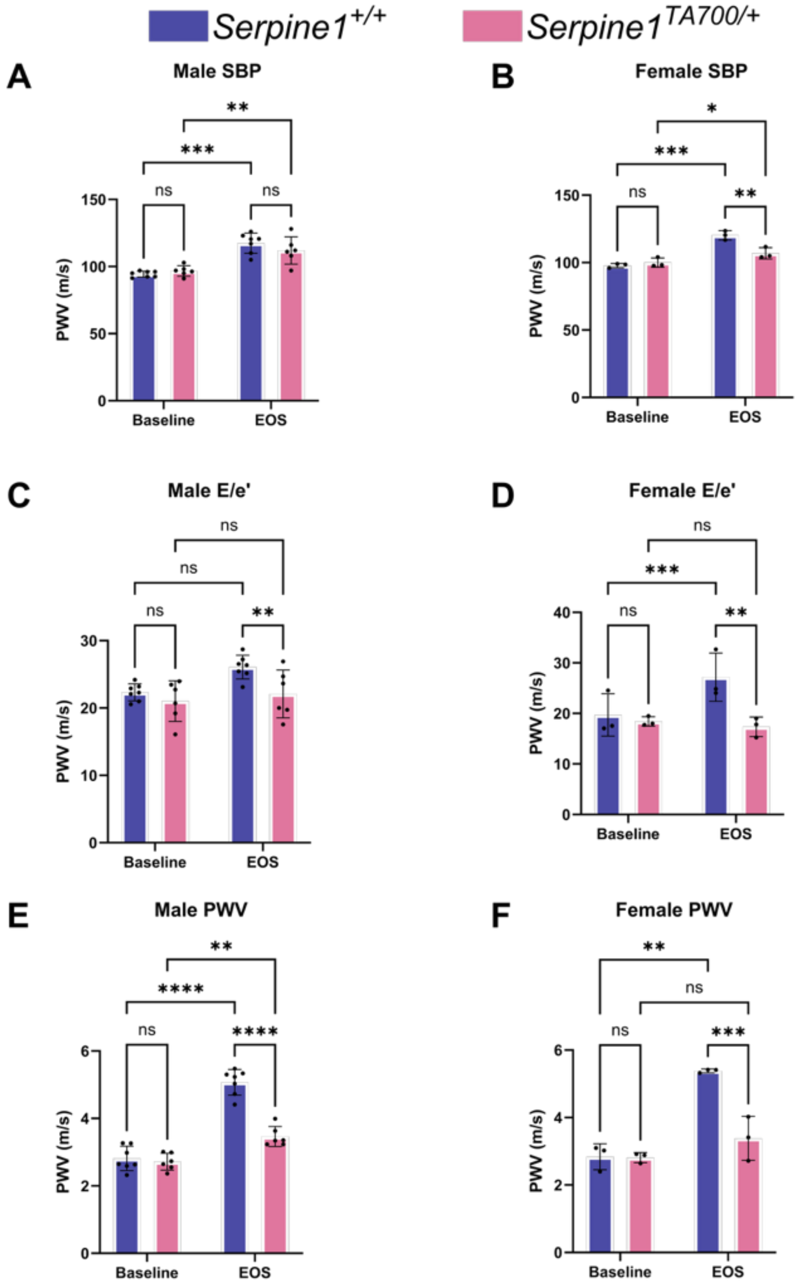

Supplemental Figure 4. Sex-separated cardiovascular physiology measurements of *Serpine1*$^{TA700/+}$ and *Serpine1*$^{+/+}$ mice. SBP measurements of (**A**) male and (**B**) female mice. E/e' measurements of (**C**) male and (**D**) female mice. PWV measurements of (**E**) male and (**F**) female mice. Statistical testing details and number of biological replicates are reported in main Figure 2.

Supplemental Figure 5.

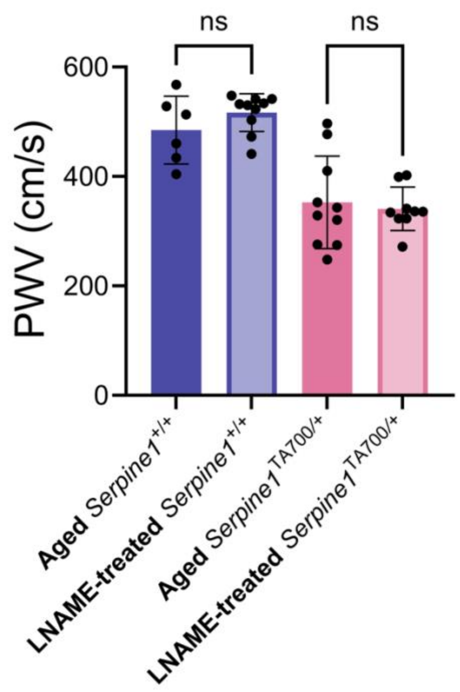

Supplemental Figure 5. PWV of aged mice and L-NAME treated mice. Comparison of aged *Serpine1*$^{+/+}$ (n=6), aged *Serpine1*$^{TA700/+}$ (n=10), L-NAME-treated *Serpine1*$^{+/+}$ (n=10), and L-NAME-treated *Serpine1*$^{TA700/+}$ mice (n=10). To determine significance, one Way ANOVA with Tukey's correction for multiple testing used.

Supplemental Figure 6.

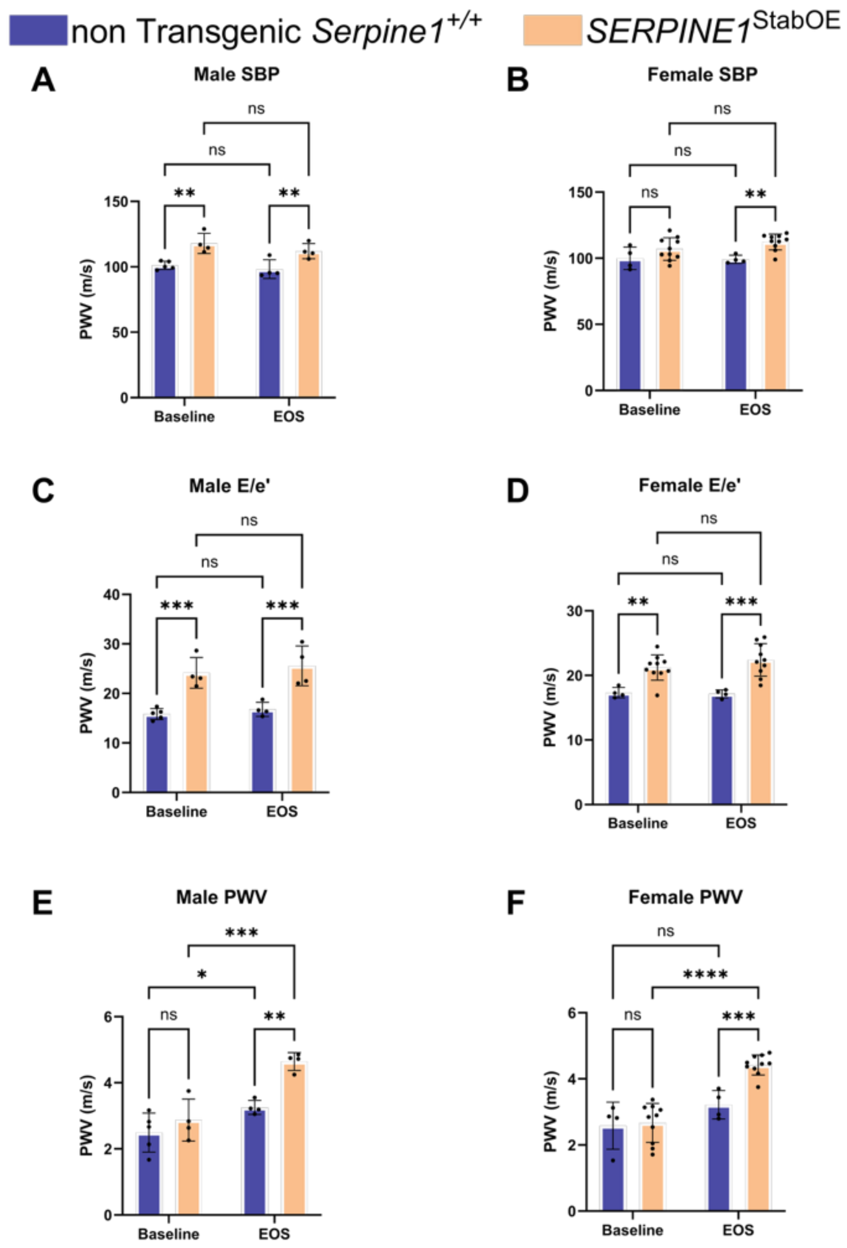

Supplemental Figure 6. Sex-separated cardiovascular physiology measurements of *SERPINE1*^{StabOE} and non-Transgenic control *Serpine1*$^{+/+}$ mice. SBP measurements of (**A**) male and (**B**) female mice. E/e' measurements of (**C**) male and (**D**) female mice. PWV measurements of (**E**) male and (**F**) female mice. Statistical testing details and number of biological replicates are reported in main Figure 3.

Supplemental Figure 7.

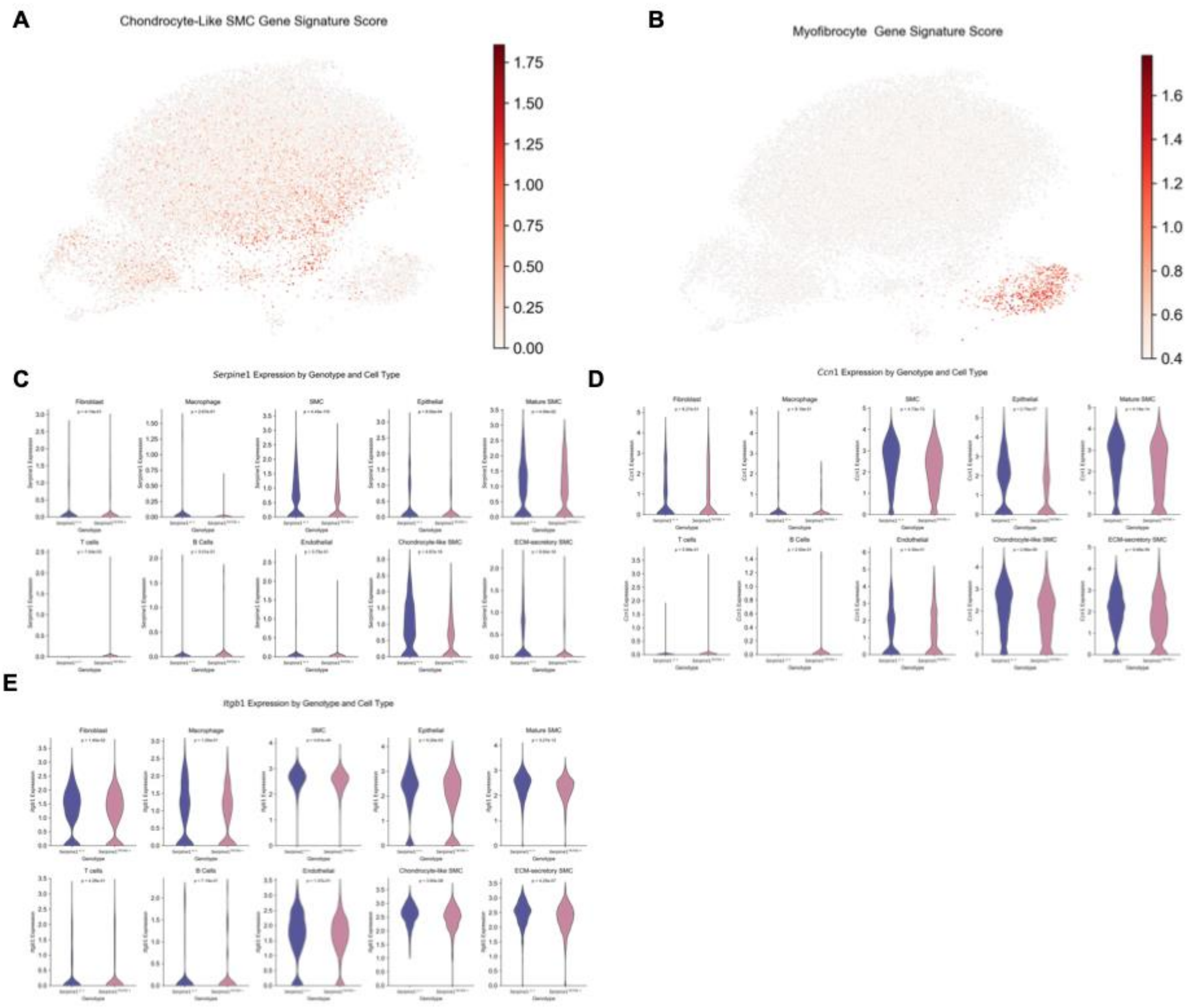

Supplemental Figure 7. Expansion of scRNA-seq findings. (**A**) UMAP plot highlighting VSMCs enriched for a chondrocyte-like smooth muscle cell (SMC) gene signature, identified using markers associated with chondrocyte-like characteristics: *Lgals3*, *Acan*, and *Tnfrsf11b*. (**B**) UMAP plot showing VSMCs enriched for an ECM-secreting SMC gene signature, defined by co-expression of VSMC and fibroblast markers: *Adamtsl1*, *Lama2*, *Fbln1*, and *Vcan*. Comparison of differences in genotypic expression of *Serpine1* (**C**), *Ccn1* (**D**), *Itgb1* separated by cell types. Certain subpanels were shown in Figure 5.

Supplemental Figure 8.

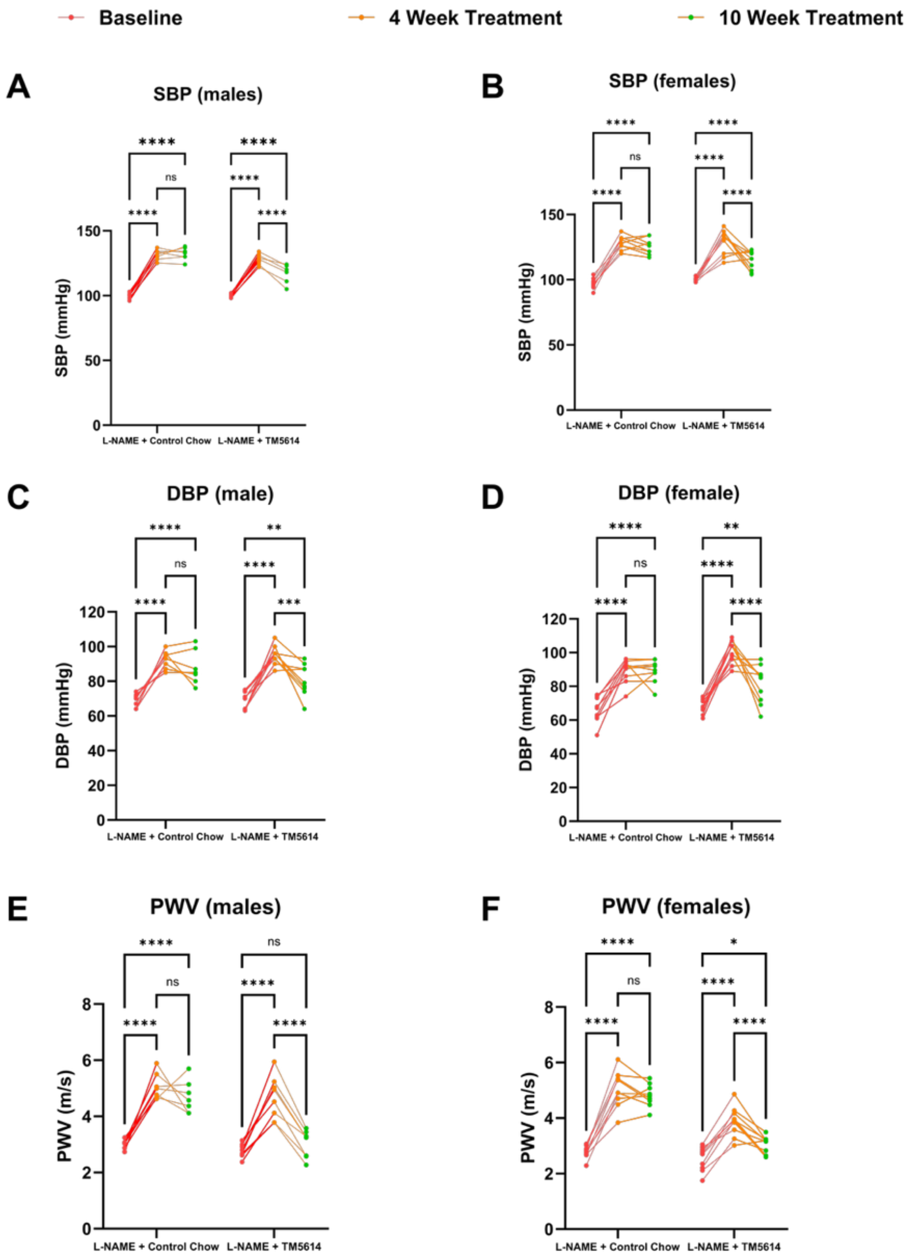

Supplemental Figure 8. Sex-separated cardiovascular physiology measurements of *Serpine1*$^{+/+}$ mice given L-NAME and PAI-1 inhibitor TM5614 or control. SBP measurements of (**A**) male and (**B**) female mice. DBP measurements of (**C**) male and (**D**) female mice. PWV measurements of (**E**) male and (**F**) female mice. Statistical testing details and number of biological replicates are reported in main Figure 7.
